# Lifting the curtain on the emergency department crisis: a multi-method reception study of *Larry Saves the Canadian Healthcare System*

**DOI:** 10.1186/s12913-023-10512-9

**Published:** 2024-01-04

**Authors:** Sara A. Kreindler, Mikayla Hunter, Graham W. Lea, Mandy Archibald, Kendra Rieger, Christina West, Shaikh Mehdi Hasan

**Affiliations:** 1 Department of Community Health Sciences, University of Manitoba, S113-750 Bannatyne Ave, Winnipeg, MB R3E 0W Canada; 2 Faculty of Education, University of Manitoba, 71 Curry Pl, Winnipeg, MB R3T 2N2 Canada; 3 College of Nursing, University of Manitoba, 99 Curry Pl, Winnipeg, MB R3T 2M6 Canada; 4 School of Nursing, Trinity Western University, 22500 University Drive, Neufeld Science Building, Langley, BC V2Y 1Y1 Canada

**Keywords:** Research-based theatre, Knowledge translation, Patient access, Patient flow, Arts-based health research

## Abstract

**Background:**

Despite growing evidence of the potential of arts-based modalities to translate knowledge and spark discussion on complex issues, applications to health policy are rare. This study explored the potential of a research-based theatrical video to increase public capacity and motivation to engage with the complex issues that make Emergency Department wait times such an intractable problem.

**Methods:**

*Larry Saves the Canadian Healthcare System* is a digital musical micro-series developed from extensive research examining system-level causes of Emergency crowding and the ineffectiveness of prevailing approaches. We released individual episodes and a revised full-length version on YouTube, using organic promotion strategies and paid advertising. We used YouTube Analytics to track views, engagement and viewer demographics, and content-analyzed viewer comments. We also conducted five university-based screenings; 92 students completed questionnaires, rating *Larry* on 16 descriptors using a 7-point Likert scale.

**Results:**

From June 2022 through May 2023, *Larry* garnered over 100,000 views (76,752 of the full-length version, 35,535 of episodes), 1329 likes, 2780 shares, and 139 comments. Views and watch time were higher among women and positively associated with age. Among YouTube comments, the predominating themes were praise for the video and criticism of the healthcare system. Many commenters applauded the show’s accuracy, humor, and/or resonance with their experience; several shared healthcare horror stories. Students overwhelmingly agreed with all positive and disagreed with all negative descriptors, and nearly unanimously deemed the video informative, thought-provoking, and entertaining. Most also affirmed that it had increased their knowledge, interest, and confidence to participate in discussions about healthcare issues. Neither gender, primary language, nor employment in healthcare predicted ratings, but graduate students and those 25+ years old evaluated the video most positively.

**Discussion:**

These findings highlight the promise of research-informed musical satire to inform and invigorate discourse on an urgent health policy problem. *Larry* has reached tens of thousands of viewers, garnered excellent feedback, and received high student ratings. Further research should directly assess educational and behavioural outcomes and explore what facilitative strategies could maximize this knowledge translation product’s potential to foster informed, impactful policy dialogue.

**Supplementary Information:**

The online version contains supplementary material available at 10.1186/s12913-023-10512-9.

## Introduction

Long Emergency Department (ED) wait times plague health systems across the world [1], but the problem is particularly severe in Canada [2, 3]. Research has traced intractable ED crowding to system-wide misalignment between service capacity and population needs, which has resulted in ever-increasing reliance on the ED [4, 5]. The ED’s role has ballooned from the care of emergencies to the investigation and management of a myriad of health problems at varying levels of acuity and complexity, compromising its ability to provide rapid, efficient care [6]. This complex healthcare challenge can be redressed only at the level of system design, by ensuring that the system as a whole is configured to provide appropriate care in appropriate locations. Unfortunately, the prospect of system redesign seems remote, especially given that Canada’s delivery system is highly devolved and fragmented [7]. Instead, local health systems rely on short-term fixes and intense day-to-day efforts to propel patients through a malformed system, with predictably disappointing results [8].

Although calls for system transformation abound in Canadian policy circles, to muster the required political will is no easy task. Without a groundswell of public support for health-system reform, governments are unlikely to defy the powerful entrenched interests and institutions that favour the status quo [9]. It is important, then, to extend dialogue beyond the closed circle of policymakers and mobilize those who could hold such policymakers accountable. Simply providing avenues for patient/public input is not enough: while patients can eloquently describe the challenges they have experienced in accessing care, the workings of the health system remain largely opaque to them. Some fundamentals of health policy remain absent from public discourse, or may appear only in oversimplified, polarized form. Although important work has been done to popularize policy ideas, levels of public interest, engagement and understanding remain insufficient to generate significant pressure for evidence-informed system change. There is a need for innovative strategies to foster a more informed, engaged citizenry, who can connect their everyday healthcare experiences to policy-level choices. This study explored the public appeal and educational potential of a non-traditional – specifically, arts-based – mode of translating knowledge on the complex of issues that make ED crowding such an intractable problem.

### Arts-based research: health and policy applications

Arts-based health research – defined as the use of arts modalities to generate and/or disseminate research findings – is a burgeoning field [10–12]. A 2012 scoping review identified 71 relevant publications [13] – leaving aside the large literatures on the use of artistic forms in health promotion (e.g., entertainment education [14]) or artistic training to enhance clinical skills [15]. Theatre is one of the most common types of art employed [13], and has been the topic of large reviews of its own [16, 17]. Arts-based knowledge translation (ABKT) is a narrower term referring to the use of the arts to communicate research findings [10]. Studies have suggested that ABKT offers important advantages over traditional modes of dissemination. An artistic presentation can make findings accessible to a broader audience, including those who might not otherwise seek out information on the topic [10, 13, 18]. Audience engagement can be significantly enhanced, both by evoking emotion and by inviting audiences into the cognitive work of interpreting a story, joke, or image [13, 18, 19]. Further, metaphor more readily communicates ambiguous, conflicting, or complex findings [10, 20, 21]. Arts-based methods are ideal for stimulating critical understanding and dialogue, including dialogue among groups that might otherwise lack a shared language [10, 22].

Despite the breadth of scholarly interest in arts-based knowledge translation, the topics to which it has been applied remain somewhat limited. Common uses of arts-based methods have been to illuminate the illness experience (and thus promote awareness and empathy on the part of providers or the public) and to transmit health information [12, 13]. In contrast, there are few published examples of ABKT on health systems and policy issues [23–26]. However, the picture is different in other fields: plays and other performances (e.g., stand-up comedy) are frequently used to increase public understanding of systemic issues ranging from institutional racism to climate change [19, 27–29].

A powerful genre for addressing such issues is satire, which can expose systemic errors in prevailing assumptions or practices by highlighting inherent absurdities and contradictions [27, 30]. One satirical research-based theatre piece (which incorporated song and movement) presented a system-level critique of Ontario’s return-to-work policies; a knowledge-translation case study highlighted several positive outcomes, but formal evaluation does not appear to have been conducted [26]. However, highly sophisticated literature on the impacts of satire exists outside the healthcare field, incorporating experimental and controlled observational studies as well as fine-grained qualitative analyses [27, 28, 31–36]. Research has found that satire can attract audiences with low initial levels of awareness/interest in the topic, especially youth [31, 32]; effectively transmit factual knowledge and influence attitudes [28, 33]; and serve as a gateway to traditional information sources, increasing topic-relevant information-seeking, attentiveness to news coverage, and assimilation of new information [34, 35]. Satire can also increase audience members’ sense of *internal political efficacy*, that is, capacity to participate in political discourse – a particularly important metric when the issues may otherwise be perceived as impenetrable or overwhelming, leading to disengagement [31, 36]. Finally, satire can disrupt prevailing power relations and help to build community, galvanizing and mobilizing those for whom its message resonates most strongly [30].

Notwithstanding their promise, research-based theatre (RbT) in general and satire in particular carry certain risks that must be assessed and managed. First, while it is demonstrably possible to develop a production that is both aesthetically satisfying and evidence-based [37], practitioners must carefully manage the tension between science and art [20, 37, 38]; indeed, this is true for arts-based knowledge translation in general [11, 21, 39]. It is not necessarily wise to privilege academic over aesthetic considerations, as audience engagement may depend heavily on a work’s aesthetic quality or entertainment value [30, 37, 38]. For example, if a research-based performance aimed at the general public is billed as a comedy, it must truly be funny; any threat to humour is also a threat to effectiveness [27, 33]. Second, there is a risk that research-based productions will “preach to the converted,” failing to attract those who do not already care about the topic [27]. Two strategies for mitigating this risk are to target performances to naturally occurring groups (e.g., school/university classes) and to aggressively market them to the public, focusing on their entertainment value. Reach (size and diversity of audience) is a highly important evaluation domain for ABKT [18, 28]. Third, satire exposes patterns and tendencies by presenting them in exaggerated form; of necessity, not all the details are to be taken literally. As such, satire – and comic presentations in general – may risk being dismissed as a mere joke (*message discounting*) [40]. Intriguingly, however, one study found that entertainment value tempered the relationship between comedy and message discounting; when a satirical sketch was appreciated as engaging and memorable, discounting was less likely to occur [33]. Furthermore, the mechanisms by which comedy may promote persuasion (e.g., increased receptiveness) may outweigh the effects of discounting [28, 33]. Nonetheless, evaluation should assess this potential negative outcome. Finally, satire can provoke defensiveness on the part of those whose power or privilege is being called into question [19]. Thus, it is important to consider its intended audience; it may not convert the individuals who hold the most influence and power to impact a problem, but rather, may stimulate a broader public to recognize and oppose the identified follies or injustices [30]. Building on what is known about the benefits and risks of satirical research-based theatre, it is important to evaluate novel applications of this genre.

### Study overview

Our study investigated the reception of a satirical musical based on extensive research examining the system-level causes of Emergency Department crowding and the ineffectiveness of prevailing solutions [4, 8]. At its core were the sobering findings of the four-province Western Canadian Patient Flow study: across jurisdictions, service offerings remained badly misaligned with population needs, resulting in persistent inefficiency, widespread use of inappropriate locations of care, and in many hospitals a perpetual state of overcapacity [5, 8]. Many managers appeared to accept, or even celebrate, the use of strenuous day-to-day flow-management strategies to compensate for the lack of rational system design [41, 42]. Study findings were disseminated in seven peer-reviewed articles and in decision-maker–oriented reports and summaries for the participating organizations, but these strategies did not make the analysis accessible and engaging to the public. The decision to pursue an arts-based approach stemmed from the realization that the topic, with its themes of misalignment, misdirected efforts, and disorganization, was ripe for satire; music and rhyme were incorporated to make content more memorable and heighten humor.

*Larry Saves the Canadian Healthcare System* follows an idealistic young policy analyst striving to get to the bottom of ED crowding. On his quest, Larry encounters poorly designed structures, dubious improvement projects, bankrupt ideas, managerial inertia, political posturing, and the ghost of Tommy Douglas (Canada’s father of Medicare). The writer/composer is a health systems researcher with experience as a playwright, songwriter and satirist (SK). The one-hour piece was originally conceived as a live theatrical performance; however, on the basis of safety considerations arising from the COVID-19 pandemic, we pivoted to video, using a green screen to enable each actor to perform separately. *Larry*’s development, which is discussed in a companion article [43], included a five-day Zoom-based workshop with feedback from a professional dramaturg and a small audience of researchers and healthcare professionals. We also sought advice from a communications professional on packaging the material for YouTube, which led us to subdivide the script-cum-screenplay into 11 micro-episodes. The full series can be viewed at https://youtu.be/U_7weVo2qV0.

The study had two components, whose methods and results are presented separately below. First, we launched *Larry* on YouTube and tracked its reach and reception amongst the general public. Second, we screened it for university students in order to evaluate its potential utility for teaching health policy topics. The inclusion of both components allowed us to gauge the reactions of a broad range of audiences under controlled as well as uncontrolled conditions.

## Methods

### Assessment of public reception

Two versions of the digital micro-series were released. In June 2022, we published each episode as a separate video, releasing these in batches in the hope of building momentum. However, we observed substantial viewer drop-off between episodes and release dates, and during the pre-show content (e.g., credits) at the start of each episode [43]. Accordingly, in February 2023 we re-released *Larry* as a full-length video, recut to start with a high-energy clip and eliminate credits between episodes. The revised version also redressed one episode that, in the writer’s hindsight, had allowed the satire to depart too far from the evidence (details in companion article) [43]. Individual episodes ranged from 4:13 to 7:32 min in length; the full-length version’s length was 57:13 min.

To promote the episodic (original) version, we e-mailed more than 50 groups and influencers likely to be sympathetic to the content, such as health coalitions, healthcare unions, social justice groups, patient associations, and individuals/organizations involved in disseminating health policy evidence. Some of the recipients publicized the playlist through their e-mail lists, newsletters and/or social media channels; unfortunately, we could not track how many did so, as some of these modes of sharing are private. We also sent press releases to local and national news organizations, highlighting the topic’s relevance to current events. The show was covered by local CBC radio and ChrisD.ca, a large independent Winnipeg news blog; one national news organization conducted an interview but did not air it. We also used e-mail and social media, including our personal Facebook, Twitter, and LinkedIn accounts and a Twitter account created for “Larry,” to circulate the videos to our own personal and professional networks.

We used YouTube advertising as the main promotional strategy for the full-length (revised) version, having exhausted our methods of non-paid promotion on the original version. We did re-contact those who had shared the previous version, but naturally, only some were willing to share what was essentially the same show a second time.

Our YouTube ad bore the slogan “The hilarious healthcare musical,” with an image of a nurse pushing a heavily bandaged patient down a hospital hallway. Based on rapid-cycle testing conducted prior to the release of the full-length version, this slogan and image garnered more clicks than options not highlighting the healthcare aspect (e.g., “Bureaucracy: the musical”). The view (click) rate for the ad was 1.72%. It is difficult to ascertain how this compares to other ads, as YouTube’s posted benchmark (10–15%) conflates ads that play automatically before a video with ads that users must click to view [44]. Anecdotal evidence suggests that a view rate of 1–3% may be typical for ads of the latter type [45].

Google Ads (the seller of YouTube advertising) gives advertisers the option of targeting ads by geography, demographics, and viewer interests as reflected in prior online behaviour. Google can access the self-reported gender and age of viewers who are signed into their Google accounts, resulting in fairly accurate assessment of these attributes [46]. However, Google may or may not make accurate inferences about viewers’ income, parental status, or level of education, all of which are often coded as “unknown.” We targeted our ads to viewers in Canada who showed relevant interests (art and theatre, news and politics, and/or healthcare) and had watched similar content (e.g., videos addressing social issues; music theatre such as Broadway or opera; satire and political humor). After the first two weeks, we explored demographic targeting, as described below.

It is important to note that the two versions differed in both content and distribution method. The revised version had the advantage of improvements made on the basis of analytics and viewer feedback, while the original version had the advantage of access to the most supportive audience (that is, members and followers of sympathetic groups and influencers). Therefore, although results will be presented separately for the two versions, it is not meaningful to compare the performance of the two distinct releases of *Larry*.

We extracted information on video views, engagement (shares, likes, comments, view duration), and viewer demographics from Google Analytics. We also conducted qualitative content analysis [47] on the comments posted by viewers. After reviewing preliminary data, two researchers (SK, MH) developed a coding scheme; its major categories included comments about the video (praise, criticism, other); comments about healthcare; and comments about politics. Coding was then led by one author (MH) and reviewed by two others (GWL, SMH), with disagreements resolved by consensus; to reduce the risk of bias, the creator of the video (SK) did not participate in this phase.

### Assessment of student reception

#### Participants

Five screenings of *Larry* were presented at the University of Manitoba: three in health-policy–related classes; two at lunchtime events for healthcare students. This convenience sample included all classes whose professors were willing to screen the video during the 2022-23 academic year. Screenings occurred between November 2022 and April 2023; owing to their timing, the first two screenings used the episodic version of the video and the last three used the full-length version. Of approximately 120 students who attended a screening, 92 completed a voluntary, anonymous questionnaire, a response rate of 77%. Participant characteristics are described in Table 1.

#### Procedure and materials

Questionnaires were offered in hard copy and/or through a QR code pointing to Survey Monkey, depending on what was most practical at each screening. As an incentive to complete the questionnaire, respondents had the chance to enter a draw for a $200 gift card; a separate sheet or webpage was used for entry forms so that names could not be linked to study responses.

The questionnaire invited students to rate the video on 16 descriptors using a 7-point Likert scale from 0 (*does not describe at all*) to 6 (*describes completely*; items appear in Table 2, full questionnaire in Additional File 1). These descriptors were adapted from prior studies of audience response to issue-based satire [28, 33]. Ten items focused on evaluation of the video, covering both informational and entertainment value (“evaluation scale,” α = 0.84; the three negative items were reverse-scored). Five items concerned the video’s influence on participants’ knowledge, attitudes, interest and confidence to participate in discussions about the healthcare system (“influence scale,” α = 0.81). The one behavioural

**Table 1:** Participant characteristics

Characteristic	N (%)
**Level**	
Undergraduate	49 (53%)
Graduate	43 (47%)
**Gender**	
Man	21 (23%)
Woman	70 (76%)
Non-binary	0 (0%)
Prefer not to say	1 (1%)
**Age band (years)**	
Under 25	41 (45%)
25–34	30 (33%)
35–44	8 (9%)
45–54	10 (11%)
55–64	3 (3%)
**Primary language**	
English	70 (76%)
Other	22 (24%)
**Has worked in healthcare**	
Yes	55 (60%)
No	36 (39%)

item, “I plan to share this video,” belonged in neither scale. We conducted descriptive analysis for individual items and for the evaluation and influence scales. We then used t-tests or ANOVA, as appropriate, to compare scale scores across demographic categories.

Participants were also invited to sign up for a qualitative interview exploring their interpretations and behavioural intentions (see Additional File 1); however, the qualitative component will be discussed in a separate article.

## Results

### Public reception: quantitative

#### Reach and engagement

The original version of *Larry* garnered 35,535 episode views, ranging from 13,615 for Episode 1 to 1257 for Episode 11. Advertising accounted for 20% of the Episode 1 views; the other episodes were not advertised directly. Collectively, the episodes received 588 likes, 22 dislikes, 48 comments, and 921 recorded shares.

As of end May 2023, the full-length version had received 76,752 views. Advertising accounted for 91% of views but only 73% of watch time, underscoring the lower duration of ad views compared to organic views. The video attracted 741 likes, 45 dislikes, 91 comments, and 1859 shares. Based on audience retention graphs for the full-length version and Episodes 1 and 11, about 2400 viewers had seen the entire show, while about 7500 had watched from the beginning to at least the end of Episode 1.

YouTube Analytics uses unpublished algorithms to determine whether audience retention at any point in a video is above or below average, taking into account the length of the video and the source of views (i.e., advertising or organic). According to YouTube Analytics (accessed in May 2023), audience retention oscillated between average, above-average, and below-average values during Episode 1; all other episodes and the full-length version showed average or above-average audience retention throughout.

#### Audience characteristics

In reviewing demographic data provided by YouTube Analytics, we noted certain anomalies. In particular, YouTube Analytics reported that 100% of Episode 2 viewers were age 55 or older, which we knew to be inaccurate, being viewers ourselves (for episodes 3–11, Google did not collect enough demographic data to report). Accordingly, the results presented below should be interpreted with caution.

YouTube Analytics estimated that, prior to the start of paid advertising, 57% of Episode 1 viewers were female, and 100% were over the age of 24, with 90% over the age of 34. During the first five weeks of advertising, YouTube Analytics described the viewing population as 55% female and 42% under the age of 24, with an additional 53% between the ages of 25 and 44.

Prior to demographic targeting of the full-length version, YouTube Analytics reported that 52% of viewers were female and 88% were over the age of 24, but views did not increase linearly with age band. However, older and female viewers accounted for a disproportionately high share of watch time, indicating that their average view duration was longer. Women over the age of 44 made up 27% of views but 46% of watch time, with women ages 65 + making the greatest contribution to watch time. People who watch an entire video are presumably more likely to share it with others, generating further views. Thus, even if YouTube’s designations are unreliable, there may be value in targeting ads to viewers that YouTube “thinks” belong to a certain demographic. Accordingly, we explored targeting the ads to women ages 45+, then 65+; naturally, this skewed the subsequent demographics of the viewing population. Increases in the share rate and average view duration were observed after targeting began; unfortunately, it is difficult to determine causality as other strategies for increasing (organic) views occurred shortly thereafter.

Not surprisingly, the majority of views came from Canada (81% for Episode 1, 93% for the full-length version). YouTube also reported views from the United States, New Zealand, and Australia, although none of these accounted for more than 0.5% of views. Viewers were spread across Canadian cities; Winnipeg, where the show was produced, accounted for 14% of Episode 1 views but only 3% of views of the full-length version.

### Public reception: qualitative

#### Content analysis

As of May 2023, the *Larry* videos had attracted 117 comments, including 71 on the full-length version and 46 on episodes, with episodes 1 and 11 being the most-commented (with 9 and 7 comments respectively). The tally included 12 replies to what another viewer had written; however, replies by the video creator were not included.

The two predominant themes were praise for the video and criticism of the healthcare system. Nearly half the comments praised the video; the majority of these did not single out a particular aspect (e.g., *“This is genius. Take it on the road”*). Comments that praised a specific aspect most commonly highlighted the video’s informational content (*“OMG nailed it*”), sometimes in conjunction with its aesthetic value.

Wow. Kudos to the musical team, this is exactly what I would expect from a musical. My mind is boggled at the extent of information provided here. How has this video not gone viral?! Sharing everywhere….

That’s the funniest thing I have seen in awhile. Yet true.

About 40% of comments addressed healthcare; most of these were general criticism (“*The system is so bad …*”), including several healthcare horror stories. A minority expressed a specific position on a healthcare issue, some congruent with the video’s message (“*The ER video got at the root – the social determinants of health…*”), others incongruent (*“It’s simple in the end. If you don’t charge for service, then you have to ration heath care”*).

While praise predominated, just over 10% of comments were critical. The greatest number of these were directed at the problematic episode mentioned earlier, which satirized the “Wild West” nature of primary care by depicting cowboy doctors flouting clinical practice guidelines. Some viewers perceived this episode as placing blame on family doctors (“*I do not appreciate the anti Primary Care sentiment of this video*”). Such comments were largely confined to the original episode version; on the revised full-length version, only one commenter mentioned guidelines and none accused the video of singling out doctors for blame. Beyond this episode, a handful of commenters disapproved of the upbeat and humorous manner in which the video presented serious issues (“*Disgustingly childish*”).

Finally, between 10 and 15% of comments mentioned politicians, federal, provincial, and/or in general; most of these appeared on the full-length version of the video. Only two such comments were supportive; most condemned politicians’ management of the healthcare system (“*our government should be ashamed in how we are treated*”).

#### Additional findings

Comments that were e-mailed to the research team, rather than posted publicly, were not included in the analysis. Of note, however, these contained some evidence of uptake by decision-makers within and beyond Manitoba. Three decision-makers not previously known to the research team arranged online meetings to discuss potential uses of *Larry*; another invited us to present at a decision-maker–oriented conference. One healthcare leader reported that they had screened some episodes at a regional Access and Flow meeting. At least one non-Manitoba professor used the video in their (political science) class, another in a journal club; others informally recommended it to their students. As well, *Larry* attracted positive coverage in *Healthcare IT Today*, *New Zealand Doctor*, and two blogs, and supportive tweets from several healthcare thought leaders.

### Student reception (quantitative)

As shown in Table 2, participants were nearly unanimous that the video was entertaining, thought-provoking, and informative (agreement ≥ 97%), and over 90% agreed it was memorable, funny, held their attention, and made research findings easy to understand. Over 85% reported that it increased their knowledge and interest in healthcare issues and got them thinking about things differently. About 80% indicated that it increased their confidence to participate in discussions about the health system and made them want to learn more; 65% planned to share the video. Less than 10% described the video as boring or hard to follow, and only 18% as hard to take seriously. Furthermore, according to the majority of participants, the terms “informative,” “memorable,” “entertaining,” and “thought-provoking” described the video “completely.”

**Table 2:** Questionnaire responses

Item	N (%)	
	Describes	Describes Completely
Entertaining	89 (97%)	50 (54%)
Held my attention	87 (95%)	45 (49%)
Informative	89 (98%)	58 (64%)
Thought-provoking	89 (97%)	50 (54%)
Funny	86 (93%)	46 (50%)
Memorable	87 (95%)	51 (55%)
Made research findings easy to understand	84 (94%)	39 (44%)
Got me thinking about things differently	81 (88%)	30 (33%)
Increased my interest in healthcare issues	80 (87%)	33 (36%)
Increased my knowledge about the issues	80 (89%)	37 (41%)
Increased my confidence to participate in discussions about the healthcare system	74 (81%)	25 (27%)
Boring	6 (7%)	3 (3%)
Hard to follow	7 (8%)	3 (3%)
Hard to take seriously	17 (18%)	2 (2%)
Made me want to learn more	72 (79%)	31 (34%)
I plan to share this video	60 (65%)	26 (28%)

* Up to 3 responses were missing per item. Percentages are calculated based on completed items

The 10-item evaluation scale had an average total score of 5.16 out of a maximum 6.00 (*SD* = 0.74, *n* = 88); the 5-item influence scale had an average score of 4.79 (*SD* = 0.88, *n* = 90). The two scales were moderately positively correlated (*r* = .39).

Neither gender, primary language, nor whether the respondent (had) worked in healthcare significantly predicted scores on either scale. Age was positively associated with evaluation scores (*F*(2, 85) = 5.67, *p* = .005): participants aged 35 + rated the video somewhat more highly than those aged 25–34, and significantly more highly than those below age 25. However, age did not predict influence scores (*F* (2, 87) = 0.67, *ns*). Likewise, graduate students evaluated the video more positively (*M* = 5.43, SD = 0.69) than did undergraduates (*M* = 4.92, *SD* = 0.71; *t*(86) = 3.43, *p* = .001), but the two groups’ influence scores were similar (t(88) = 1.06, *ns*).

## Discussion

### Public reception

Collectively, the videos garnered over 100,000 views; this degree of reach is impressive, especially considering that the target audience was limited to Canada. Thus, YouTube proved an effective platform for reaching a large and diverse audience. Although YouTube audiences are notoriously fickle, and only a minority of viewers watched the whole hour-long show, the number that did remains greater than the number that would have been able to attend a live performance. Promotional strategies were essential to make *Larry* stand out among the over 800 million videos on YouTube [48]; promotion through sympathetic groups/influencers and paid advertising were both important, affording access to different audiences. The video appeared to be most popular among older viewers and women, groups that may have the greatest interest in musicals and/or in healthcare issues.

*Larry* garnered high praise from viewers, reflecting both its entertainment value and its encapsulation of health-system issues. While the satirical content of one episode (later amended) drew critiques, and satire itself is not a genre for everyone, overall, audiences seemed to regard the satirical tone as appropriate and welcome. The video also stimulated many to share their frustrations with the healthcare system. However, YouTube comments do not contain enough information to gauge what viewers actually learned from the show and whether/how their attitudes or behavioural intentions may have changed. We suspect that healthcare-related and political comments largely reflected pre-existing attitudes; and behavioural intentions (beyond sharing the video) were not mentioned. Moreover, a comment board does not foster interaction to the same extent as a post-show facilitated discussion, a hallmark of research-based theatre presentations [24, 38]. Further research would be needed to examine potential mechanisms of influence. In addition, it would be desirable to test *Larry* as a conversation-starter for facilitated dialogue among patients, providers, and policymakers.

YouTube analytics and viewer comments offer a wealth of information about how a video is received. To benefit from this, it important for video creators to be able to revise their initial offerings. We discovered that meaningful revisions were possible despite financial and contractual constraints that precluded re-shooting of scenes. In future, it would be ideal to start with a “soft launch,” keeping the video unlisted while exposing it to a small audience through YouTube ads and private screenings. Viewer comments could then be used to inform timely revisions, allowing intensive promotional strategies to be reserved for the strongest possible product.

We are unaware of any prior research on the use of YouTube to disseminate research-based theatre. However, this mode of distribution may become increasingly common now that the pandemic has brought all forms of digital communication to the fore. Our findings may be helpful to future creators.

#### Limitations

This sub-study enabled us to investigate the video’s reach and reception naturalistically. However, as noted above, the available information about YouTube viewers and their reactions is limited and not always reliable (i.e., demographic breakdowns from YouTube Analytics may not be accurate). As well, it is difficult to gauge how many viewers found the content valuable; shares, likes, and positive comments suggest a minimum number, but sharing that occurs outside of YouTube (e.g., by forwarding an e-mail) is not tracked, and viewer opinions are not collected systematically. Fortunately, we were able to compensate for these limitations to some extent by also examining student reception.

### Student reception

Questionnaire results indicated a highly positive response from university students in health-related classes. The great majority praised its entertainment and informational value, and indicated that it had positively impacted their knowledge, thinking, and confidence to participate in discussions about healthcare. Only a small minority thought that it was boring or hard to follow, or even that its comedic style made it hard to take seriously (indeed, a few appear to have given these ratings as part of a positive response set; that is, they expressed agreement with all items, positive and negative alike, presumably rushing through the questionnaire). These findings are congruent with studies showing that research-informed satire can have high audience appeal, and that message discounting may not occur if the piece is perceived as effective entertainment [28, 33].

Results were consistent across demographic categories, but older and graduate students gave especially favourable ratings. Perhaps their greater knowledge and/or experience of health policy issues increased their enjoyment of the satire; older audiences might also be more receptive to a retro-pastiche musical style. Nonetheless, the high overall evaluation scores across a generally young sample demonstrate that *Larry*’s appeal is not limited to older adults. We also note that owing to the timing of screenings, all graduate students saw the full-length version of the video, which was also associated with higher evaluation scores; we were unable to disentangle the effects of these two variables. Whereas older and graduate students evaluated the video more positively, they did not report greater influence on their thinking, knowledge, or confidence. For some, the video may have confirmed their existing knowledge and attitudes.

Interestingly, neither gender nor having worked in healthcare predicted ratings, although both these variables appeared to predict interest in the YouTube video (as did older age, a consistent finding across both sub-studies). The video was also rated equally highly by students whose primary language was or was not English. Overall, findings suggest that the video would be appreciated by a wide range of university students taking classes related to healthcare.

#### Limitations

Several limitations should be noted. One is the self-report nature of the data; we cannot confirm whether or what participants actually learned from the video, or even whether those who expressed an intention to share it actually did so. Social desirability considerations may also bias responses towards the positive on any feedback survey. However, the findings are promising and merit further research examining educational and behavioural outcomes.

Another limitation is the imperfect response rate; it is possible that students who did not enjoy the video were less likely to complete a questionnaire. Meanwhile, some screenings had technical glitches such as the picture going out of sync with the audio or occasionally disappearing from the screen, which may have dampened some ratings. We also note that, owing to an error, participants at one screening (*n* = 17) completed a Likert scale with 6 instead of 7 points, meaning that it lacked a neutral point. For analyses of summed scores, we recalibrated these participants’ responses on a 7-point scale. Had we instead excluded these participants, the mean evaluation score would have been slightly higher and the mean influence score virtually identical. Thus, although we cannot determine how the 6-point scale may have affected responses, its inclusion seems unlikely to have biased our findings in a positive direction.

Finally, we were able to arrange screenings in a limited number of classes, none of which were part of undergraduate health professional education. However, the sample did include many students who (had) worked in the health system. The finding that the content resonated with healthcare workers and graduate students is a strength; on the other hand, the strong representation of these groups is also a limitation, as *Larry*’s original intent was to introduce ideas to non-experts. It would be beneficial for further research to include university students in non-health-related courses, and even high school students. Whereas uptake of the video is likely to remain highest among instructors teaching healthcare topics, broader use would better support the goal of familiarizing new audiences with health policy fundamentals, in particular the systemic causes of Emergency Department crowding. We also note that, as three quarters of participants had English as their first language, some caution is warranted in generalizing the findings to culturally and linguistically diverse audiences.

### General discussion

This study reveals how arts-based knowledge translation can fruitfully address a novel – some might even say unlikely – topic. Research-based theatre has been used to unpack policy dilemmas regarding genetic testing [24, 25] and to critique health policies [26] but not, to our knowledge, to explicate a multicausal health-system problem. Furthermore, at least within the health domain, the most common mechanism of research-based theatre seems to be the presentation of human stories to evoke empathy and deepen understanding of patient (and/or caregiver) experiences [13, 24, 49–51]. *Larry*, in contrast, sought to translate a much more abstract set of ideas, which called for a different artistic approach. While the video did dramatize the plight of patients to some extent, it focused more on mining structural and organizational dysfunction for comedy. The positive audience response suggests that the possibilities for RbT may be broader than previously imagined. However, our findings also raise the question of what topics might *not* be suitable for RbT, or indeed for ABKT of any kind, and why. This question has seemingly been left to artistic intuition, but merits scholarly exploration.

While *Larry* is unique in terms of its subject matter and perhaps its degree of irreverent zaniness, it still fits comfortably within the spectrum of research-based theatre delineated by Beck and colleagues [37]. Their spectrum is based on two axes: the *research continuum* (ranging from casual inquiry to systematic research) and the *performance continuum* (ranging from “closed performances” exploring source data to public-facing “aesthetic performances” prioritizing aesthetic value over literal representation). As an RbT product, *Larry* positioned itself in the relatively unfrequented top-left corner of the spectrum, being an aesthetic performance with systematic research as its primary source. Some have suggested that RbT typically subordinates aesthetics to pragmatic aims [52]; in contrast, some practitioners defend the opposite approach [11]. For example, Schneider and colleagues (2014) argued that to generate drama capable of engaging a wide audience required a “creative and imaginative process… unconstrained by ‘worthy’ or didactic aims.” (p. 62) [53]. This was very much the philosophy that informed *Larry*. Our study adds to the evidence that research-based theatre can “square the circle,” offering both educational and entertainment value.

Nonetheless, *Larry* was not immune to the well-known tension between aesthetic and educational goals [11, 20, 37–39]. As in the case of its problematic episode, artistic license may increase the risk of potentially misleading representations. We also recognize a more subtle source of dissonance between scientific and aesthetic aims: As Archibald and colleagues (2012) have noted, whereas traditional conceptions of knowledge translation emphasize precise communication, “ambiguity and the individual nature of interpretation are fundamental components of engagement with the arts” (p. 317) [10]. In their classification schema for ABKT approaches, these authors included a continuum from precision to ambiguity of key messages, noting that ambiguity may be desirable for stimulating critical reflection and dialogue. *Larry* is not inherently ambiguous in the way of abstract art or dance; its key messages are in fact articulated by characters. However, aesthetic goals demanded that such messages not be belaboured didactically but embodied in a way that left space for interpretation; thus, they might have been easily apprehended by some viewers but missed or misconstrued by others. This reflection suggests that, in general, ambiguity increases as a research-based theatre piece approaches the aesthetic end of Beck and colleagues’ (2011) performance continuum [37]. We view this not as an argument against aesthetic performances but rather in favour of the pluralistic combination of knowledge translation approaches, such that each contributes its distinctive affordances [52]. ABKT in general may not be the most efficient means of conveying factual knowledge [54]; its unique value lies in such areas as the communication of findings that cannot be distilled into simple propositional statements, and the enhancement of audience interest and critical engagement [13, 20, 38].

## Conclusion

These preliminary findings highlight the promise of research-informed musical satire as an educational tool for university students and the public. *Larry* has reached tens of thousands of viewers, garnered outstanding audience feedback, and received high ratings across a wide range of students taking health policy classes. Of course, the reach and reception of this particular product are a function of myriad factors, not all of which we can identify; thus, caution should be applied in transferring findings to other contexts. Nonetheless, this study presents an informative example of arts-based knowledge translation’s possibilities and challenges. It is, to our knowledge, the first study of ABKT in relation to healthcare delays, and provides encouraging indications that artistic approaches can contribute to breaking the policy deadlock that prevents systemic causes from being addressed.

Interview findings will shed further light on students’ perspectives, and future studies should directly examine educational outcomes in both student and general audiences. As well, since the ultimate goal is to foster better-informed and more impactful dialogue, research should explore how *Larry* might be used as part of a process whereby public frustrations can be channelled into meaningful engagement in system change.

### Electronic supplementary material

Below is the link to the electronic supplementary material.

**Supplementary Material 1:** Research instruments

## Data Availability

The datasets supporting of the conclusions of this article are not in a repository, but are available from the corresponding author on request. The video itself is available at https://youtu.be/U_7weVo2qV0.
