# Supplementary material for: Lifting the curtain on the emergency department crisis: a multi-method reception study of Larry Saves the Canadian Healthcare System
Source: BMC Health Serv Res. 2024 Jan 4;24:13. doi: 10.1186/s12913-023-10512-9 (PMC10765753; doi:10.1186/s12913-023-10512-9)
Supplement: Supplementary file 1 — Supplementary Material 1: Research instruments [file 12913_2023_10512_MOESM1_ESM.docx]

**Questionnaire**

Here are some words and phrases that people might use to describe a video. For each one, please rate how well it describes the video you saw today, by circling a number from 0 (*does not describe at all*) to 6 (*describes completely*). There are no right or wrong answers.

|  | **How well does this word or phrase describe**  **the video you saw?** |
| --- | --- |
|  | Not at all------------------------------------------Completely |
| Entertaining | 0 1 2 3 4 5 6 |
| Held my attention | 0 1 2 3 4 5 6 |
| Informative | 0 1 2 3 4 5 6 |
| Thought-provoking | 0 1 2 3 4 5 6 |
| Funny | 0 1 2 3 4 5 6 |
| Memorable | 0 1 2 3 4 5 6 |
| Made research findings easy to understand | 0 1 2 3 4 5 6 |
| Got me thinking about things differently | 0 1 2 3 4 5 6 |
| Increased my interest in healthcare issues | 0 1 2 3 4 5 6 |
| Increased my knowledge about the issues | 0 1 2 3 4 5 6 |
| Increased my confidence to participate in discussions about the healthcare system | 0 1 2 3 4 5 6 |
| Boring | 0 1 2 3 4 5 6 |
| Hard to follow | 0 1 2 3 4 5 6 |
| Hard to take seriously | 0 1 2 3 4 5 6 |
| Made me want to learn more | 0 1 2 3 4 5 6 |
| I plan to share this video | 0 1 2 3 4 5 6 |

Comments

**About you (*please circle*)**

Gender: man

woman

non-binary (i.e., gender fluid, gender queer, agender, non-binary)

or please specify: ___________________________________

prefer not to answer

Age group: under 25 25-34 35-44 45-54 55-64 65+

Is English your primary language? yes no

Do you work/have you worked in the healthcare system? yes no

Have you seen this video before? yes no

**THANK YOU!**

*If you have additional comments or suggestions about the video, please feel free to use the space below, and/or sign up for an interview. We truly appreciate all feedback.*

**Interview Guide**

1. What do you think the show was about?
2. Probe (if needed): What do you think it was trying to say about the healthcare system?What resonated the most with you? What resonated the least?
   - Probe (if needed): What most (least) fit with your experience of the healthcare system?
   - Probe (as needed): How do you see the issues in this video connecting to issues in healthcare today?
   - Probe: Were there any characters or situations with which you could identify? (How so?)
3. What parts of the show did you enjoy most? Least? (Why?)
4. Did you gain any new knowledge or insight as a result of seeing the show? (How so?)
5. Did the show challenge your perspective on the issues in any way? (How so?)
6. Was there anything about the show that bothered you? (How so?)
7. Could you comment on how the visuals enhanced or detracted from the content?
8. Can you describe anything that you've done or plan to do as a result of seeing the show?
   - (If prompted, e.g., sharing the video, discussing the issues with others, seeking out information, getting involved in advocacy about the healthcare system)
   - Probe if a healthcare student/provider: How, if at all, might it influence your practice?
   - Probe: How did the show make you feel about the prospect of achieving change in the healthcare system?
9. Who, if anyone, do you think should see this show? (Who should be its target audience?)
   - (Probe if needed: Let's say we have a limited advertising budget – who do you think would get the most out of the show?)
10. How effective do you think the show would be as live theatre instead of an online video?
    - How about as a one-hour YouTube video vs. a series of episodes?
11. May I ask which age group you fall into: Would it be under 25, 25-34, 35-44, 45-54, 55-64, or over 65?
12. And for the transcript,* how would you like us to record your gender?
13. That brings me to the end of my questions. Is there anything else you'd like us to know?

(* or "for our records" if participant did not consent to be recorded)
